# Views of Japanese medical students on the work-life balance of female physicians

**DOI:** 10.5116/ijme.5907.0d44

**Published:** 2017-05-11

**Authors:** Keiko Takahashi, Tomoni Nin, Megumi Akano, Yukiko Hasuike, Hiroko Iijima, Keiichirou Suzuki

**Affiliations:** 1 Medical Education Center, Hyogo College of Medicine, Japan; 2 Department of Otorhinolaryngology-Head and Neck Surgery, Hyogo College of Medicine, Japan; 3 General Affairs department, Hyogo College of Medicine, Japan; 4 Department of Internal Medicine, Division of Kidney and Dialysis, Hyogo College of Medicine, Japan; 5 Division of Hepatobiliary and Pancreatic disease, Department of Internal Medicine, Hyogo College of Medicine, Japan

**Keywords:** Medical students, work-life balance, gender equality, diversity, Japan

## Abstract

**Objectives:**

To survey medical students on their ideas of future work-life balance and discuss topics for next-generation medical education.

**Methods:**

First-year (n=372, 34.9% female) and sixth-year medical students (n=311, 44.1% female) responded to a questionnaire on future self, marriage and childcare, and gender differences at the workplace. Responses were compared between academic years and gender. Responses were evaluated by gender and academic year using the Mann-Whitney U test.  Significance was set at p<0.01.

**Results:**

The first-year and sixth-year students, regardless of gender, had different views on gender-related favorable treatment at workplaces {U=13464, p=0.000 (first-year), U=10407, p=0.000 (sixth-year)}. A greater percentage of female students would choose career options based on the possibility of marriage and childbirth {U=10689, p=0.000 (first-year), U=10930, p=0.000 (sixth-year)}. Among first-year students, a greater percentage of female students expected to work part-time. Also among first-year students, greater percentages of female students expected to work part-time or leave their jobs temporarily while raising their children. Compared with first-year male students, first-year female students expected to undertake larger portions of the childcare and housework burden than their partners. However, gender differences in work-life balance and childcare leave vanished in the sixth-year students.

**Conclusions:**

Female medical students accepted childcare and housework burdens as inevitable; the work environment they choose might affect their career development. While support from male partners and institutions must be increased, voluntary actions and change in mentality of female students need to be promoted through medical education to prevent them from waiting passively for the situation to change.

## Introduction

Japanese medical schools offer a six-year program. Japanese medical schools require no work experience of their students before admission. Therefore, most Japanese medical students have never previously worked. There are 80 medical schools in Japan as of 2015. The maximum capacity of the medical schools is 9,079; female students accounted for over 40% of these students in 2015 and this percentage is increasing.^1^ However, only 17.4% of practicing physicians are female.^2^ Considering that the number of physicians needed exceeds the number of current practicing physicians in Japan,^2^ the working environment for physicians needs to be improved in order to encourage female physicians to stay in active practice. A survey conducted by the Ministry of Health, Labour and Welfare (MHLW) of Japan showed a temporary decrease in the female labor participation rate between the ages of 25 and 40,^3^ suggesting that women may leave their jobs temporarily due to marriage, childbirth, and/or childcare.  Previous studies found that female doctors’ career improvement was susceptible to family influences and was also susceptible to national culture.^4^^,^^5^ While environmental support to prevent a decrease in the female labor participation rate regardless of age would be ideal, not all women wish to work throughout their lives or get married and have children. Stereotypical support will not increase the overall female labor participation rate. In addition, reports on obstacles to women's career development have been reported not only from Japan but also from other countries.^6^ Our medical school provides students with an opportunity to consider their future work-life balance through a program related to gender equality in their fourth year just before they start their clinical training. However, the students’ perception of the program is unknown. This study explored the environment and medical education system necessary to support both male and female physicians in developing their careers through social partnerships. We conducted a survey of our medical students’ ideas about work-life balance and their lives and careers after they finish the current medical education program and become physicians.

## Methods

The 12-item multiple-choice questionnaire included questions about the medical student’s views on his/her future self, marriage and childcare, recognition of gender differences at workplaces, and aspirations for the future (see Appendix). In this questionnaire, we tried to grasp the factors preventing female doctors from continuing to work.

### Study design and participants

The study protocol and questionnaire survey were reviewed and approved by the Hyogo College of Medicine Ethics Committee (Approval No. 1465). The questionnaire survey was distributed to 372 first-year students (242 males and 130 females) and 311 sixth-year students (174 males and 137 females) who were enrolled at the School of Medicine, Hyogo College of Medicine between 2015 and 2017.

### Instrument

The questionnaire focused on factors that would promote or hinder their future selves as physicians to continue to work. The questionnaire also gave the medical students the opportunity to think about their own future with regard to their work life and personal life. The questionnaire was administered during class hours. Verbal and written explanations about the questionnaire were given to the medical students. The students were informed about the purpose of the questionnaire and that filling out the questionnaire was voluntary. Written informed consent was obtained from each participant. It was explained to the students that there was no disadvantage of refusing to participate in the questionnaire survey. After the students filled out the questionnaire, it was explained to the students that they could still decline to participate in the survey. No student refused to fill out the questionnaire. The questionnaire was distributed to first-year medical students during their orientation immediately after their admission, and it was distributed to sixth-year medical students after they had completed all of their courses and clinical training, and immediately before their graduation.

### Procedure

The collected questionnaires and data were strictly kept by one of the authors in a locked storage cabinet. The personal computer that was used to process the data, was used exclusively for this research project and was password-protected so that only that researcher could access it, and the computer was used offline. The original questionnaire and summary data shall be kept for 10 years after publication of the research results. After the storage deadline, the data file will be erased and the paper medium will be pulverized. The questionnaire results were entered into a database, and tabulated by academic year and gender.

### Statistical analysis

The responses to each question were compared by gender among students in the same academic year, and by academic year among students in the same gender using the Mann-Whitney U test.  Statistical significance was set at p<0.01. SPSS version 20 was used for the statistical analysis.

**Table 1.1:** Aggregate results on the questions about the work environment (Question 9)

Question 9. How do you think male physicians and female physicians are treated at the workplace?	Sixth year (n=311)		First year (n=372)	
	Female (n=137) n(%)	Male (n=174) n(%)	Female (n=130) n(%)	Male (n=242) n(%)
Equally	32 (23.4)	74 (42.5)	15 (11.5)	66 (27.3)
Female favorably	5 (3.6)	13 (7.5)	3 (2.3)	7 (2.9)
Male favorably	33 (24.1)	14 (8.0)	34 (26.2)	40 (16.5)
Depend on clinical department	57 (41.6)	48 (27.6)	39 (30.0)	68 (28.1)
I don’t know	10 (7.3)	25 (14.3)	39 (30.0)	61(25.2)
Total	137 (100)	174 (100)	130 (100)	242 (100)

Mann-Whitney U test between gender of same year: p= 0.045 (Sixth year), p= 0.027(First year)

Mann-Whitney U test between academic year of same gender: p=0.000 (Female), p=0.000 (Male)

**Table 1.2:** Aggregate results on the questions about the work environment (Question 10)

Question 10. Do you think that the support system for parents having/raising children is different among the specialty fields?	Sixth year (n=311)		First year (n=372)	
	Female (n=137) n(%)	Male (n=174) n(%)	Female (n=130) n(%)	Male (n=242) n(%)
Yes	115 (83.9)	83 (47.7)	71 (54.6)	94 (38.8)
No	15 (10.9)	88 (50.6)	59 (45.4)	148 (61.2)
Other	7 (5.1)	3 (1.7)	0 (0)	0 (0)
Total	137 (100)	174 (100)	130 (100)	242 (100)

Mann-Whitney U test between gender of same year: p=0.000 (Sixth year), p=0.003(First year)

Mann-Whitney U test between academic year of same gender: p=0.000 (Female), p=0.114 (Male)

## Results

While there were no differences in the types of future work the medical students wished to have between genders or academic years, the manner of intended work involvement was different between male and female students even in the first year. A greater percentage of female students expected

to work part-time (first-year female vs. first-year male: 14.3% vs. 2.3%, sixth-year female vs. sixth-year male: 13.7% vs. 0.8%). Among the female students, community healthcare (sixth-year and first-year: 44.3% and 61.1%) was the most common interest, followed by working at a medical educational institution (sixth-year and first-year: 24.3% and 11.5%) and then placing family first (sixth-year and first-year: 11.4% and 6.1%).

**Table 2.1:** Aggregate results on the questions about gender difference in leaving one’s job (Question 11)

Question 11. What would be the reason you leave your job in the future?	Sixth year (n=311)		First year (n=372)	
	Female (n=137) n(%)	Male (n=174) n(%)	Female (n=130) n(%)	Male (n=242) n(%)
Marriage	9 (6.6)	10 (5.7)	1 (0.8)	5 (2.1)
Pregnancy (yourself or your partner)	47 (34.3)	5 (2.9)	39 (30.0)	2 (0.8)
Childbirth	37 (27.0)	9 (5.2)	29 (22.3)	9 (3.8)
Nursing care	11 (8.0)	68 (39.1)	17 (13.1)	101 (41.7)
Retirement	29 (21.2)	71 (41.0)	37 (28.6)	122 (50.4)
Other	4 (2.9)	11 (6.1)	7 (5.2)	3 (1.2)
Total	137 (100)	174 (100)	130 (100)	242 (100)

Mann-Whitney U test between gender of same year: p= 0.045 (Sixth year), p= 0.027(First year)

Mann-Whitney U test between academic year of same gender: p=0.000 (Female), p=0.000 (Male)

**Table 2.2:** Aggregate results on the questions about gender difference in leaving one’s job (Question 12)

Question 12. What is the most important factor for female physi-cians to continue working?	Sixth year (n=311)		First year (n=372)	
	Female (n=137) n(%)	Male (n=174) n(%)	Female (n=130) n(%)	Male (n=242) n(%)
Childcare environment	36 (26.3)	45 (25.9)	37 (28.5)	66 (27.3)
Work environment	66 (48.2)	70 (40.2)	50 (38.5)	110 (45.5)
Partner’s understanding	23 (16.8)	42 (24.1)	29 (22.3)	54 (22.3)
Determination / effort	7 (5.1)	13 (7.5)	13 (10.0)	12 (5.0)
Other	5 (3.6)	4 (2.3)	1 (0.8)	0 (0)
Total	137 (100)	174 (100)	130 (100)	242 (100)

Mann-Whitney U test between gender of same year: p=0.000 (Sixth year), p=0.003(First year)

Mann-Whitney U test between academic year of same gender: p=0.000 (Female), p=0.114 (Male)

Female students preferred a different work arrangement than male students even in the first year; a greater percentage of female students considered working part-time or temporarily leaving their jobs after they had children (first-year female vs. first-year male: 70.8% vs. 15.3%, sixth-year female vs. sixth-year male: 54.5% vs. 6.4%). The female students had higher expected childcare and housework burden than the male students among the first-year students (first-year female vs. first-year male: mean percentage of childcare burden, 42.3% vs. 2.9%; mean percentage of housework burden, 42.4% vs. 3.7%). The idea of work-life balance was different between the first-year and the sixth-year students regardless of gender. The gender difference in expected childcare burden noted in the first-year students was almost absent in the sixth-year students. The same could be said about taking childcare leave. As for expectations from their partner, a greater percentage of male students wished that their partners would leave their jobs after marriage or childbearing and concentrate on doing housework and providing childcare (first-year male 81.0% and sixth-year male 69.0%).

Regarding the work environment, gender-related favorable treatment at the workplace was recognized differently between the first-year and the sixth-year students in both genders {U=13464, p=0.000 (first-year); U=10407, p=0.000 (sixth-year)} (Table 1.1 and 1.2). A greater percentage of female students would choose career options based on the possibility of marriage and childbirth {U=10689, p=0.000 (first-year); U=10930, p=0.000 (sixth-year)}.  Regarding leaving one’s job in the future (Table 2.1 and 2.2), possible reasons to leave one’s job were different between the male and female students even in the first year. A greater percentage of female students responded that they would leave their jobs because of pregnancy / childbirth (first-year female vs. first-year male: 52.3% vs. 4.6%, p=0.000; sixth-year female vs. sixth-year male: 61.3% vs. 8.1%, p=0.000).

## Discussions

This study compared the change over time and gender differences in consideration of female physicians staying in the workforce. It was based on a questionnaire survey that was administered to first-year medical students right after their admission and sixth-year students immediately before their graduation.

Female students wished to continue working despite the expected change in lifestyle over time but felt that they might need to change their work arrangement after they became pregnant and had a child. On the other hand, the male students were accepting of men taking childcare leave but tended to expect their partners to stay at home. Female students perceived gender-based favorable treatment in the current clinical environment while male students were unaware of it.

Medical students are a group of people who wish to become physicians. Most of them will perform clinical work after getting a medical license although the work arrangements may be different among individuals. The current participants also said that they would become a medical doctor regardless of academic year or gender. However, the specific image of their future self-differed between male and female students even in the first year. Many female students responded that they would work part-time while few male students did so in a previous survey of university students in Japan.^7^  It has been reported that female doctors tend to select part-time work even in other countries.^4^^,^^8^ According to the 2014 MHLW statistics, 56.7% of female workers in Japan are temporary employees.^9^ The top reasons why women chose temporary employment included flexible working hours, family reasons such as childcare and nursing care, and short commuting hours.^7^ Becoming free of restrictions from housework/childcare/nursing care was the reason for wishing to switch from temporary to full-time employment given by 16.5% of female workers, while less than 1% of male workers gave it as the reason.^9^ The statistics clearly show the reality in which women accept more family-related burden of housework, childcare, and nursing care compared with men. In fact, Japanese men are involved in housework or childcare for a shorter period of time compared with men in other economically advanced countries.^7^ Our survey also showed that a greater percentage of female medical students even in their first year would customarily accept family-related burdens such as childcare and housework as in other professions. However, the gender difference in the idea of housework sharing became smaller among the sixth-year students who experienced some clinical training work. The survey conducted in Japan in 1999 when the Basic Act for a Gender-equal Society was enforced identified the presence of a concept of fixed gender roles of “Men work, women stay at home”.^3^ The Japanese conventional wisdom then was that women were inevitably responsible for housework and childcare. The gender difference in housework burden may be disappearing because the present male and female medical students who had been raised by their parents in the gender-equal generation have matured as members of the society with budding professionalism as physicians. As in Japan, there are reports that women's career advancement is influenced by the culture of the country,^4^ and there is a high possibility that women's employment support is a problem that will be addressed internationally.

The workplace environment in Japan affects the proportion of men taking childcare leave. While about 80% to 90% of men take childcare leave in workplaces with many female workers, less than 3% of men do so in workplaces where few women work.^7^ To the general question about male physicians taking childcare leave, both male and female sixth-year students said that male physicians should take a leave as their legitimate right or if a substitute physician is available. Similar to their responses to the question about childcare and housework burden, male students wished that their partners would leave their jobs after they were married or had a child regardless of academic year when the question was about their personal future. Less than half of the male students said that they would take childcare leave themselves. According to a survey conducted by the Japan Medical Association, the percentage of female physicians taking childcare leave is below the average percentage of female workers in general, which is over 90%. On the other hand, about 2% to 3% of male physicians take childcare leave while less than 2% of male workers in general do so on average in Japan.^10^ Although it has been reported in a survey of medical students in the Netherlands that male students expected that having a family would affect their partners’ careers,^4^ it was reported that when female undergraduate students in science were surveyed about going on to graduate school, they considered the necessity of abandoning having a family;^11^ therefore, this was not limited to medical students. Many female doctors were satisfied with their work situation in studies conducted in Japan and other countries, but experienced difficulty in maintaining work-life balance.^12^ It was also reported that lack of maintenance in enhancing doctor's work-work balance hurts the physician's health and there was a latent concern about safe medical provision to patients.^13^ Compared with other economically advanced countries, it is difficult for both male and female physicians to take childcare leave in Japan. Although there is a lack of physicians and the concept of childcare leave at workplaces is relatively new, physicians in Japan may be hesitant to take childcare leave because they are afraid to lag behind in their career development due to a long-term leave since the career support system at workplaces is inadequate.

The responses to the questions about work environment were different between the first-year and the sixth-year students regardless of gender. The clinical training experience may have been reflected in the responses of the sixth-year students. Female students seemed to feel that “there would be gender-related favorable treatment depending on the clinical department or the medical office.” Many female students consider their career path based on the possibility of marriage and childcare in the future before they actually start out as physicians. A greater percentage of female sixth-year students may be seeing marriage and childcare as important factors influencing their choice of future career path compared with their first-year counterparts. There was a gender difference in the reasons for leaving the job even in the first year. Greater percentages of female students gave “marriage” and “childbirth” as the reasons. This trend has also been noted in other countries. International surveys show that there are gender differences in the way physicians choose their specialty or work arrangement.^14^^,^^15^

Childbirth may be the largest obstacle for women to continue working. Men and women are equally entitled to work, but only women can give birth. Women need to accept gender differences when it comes to childbirth. Our survey showed that the female students recognized their environment (e.g., workplace, childcare, partner), rather than their determination or effort, as an obstacle for women to continue working. Female physicians have a lower re-hospitalization rate of patients in their charge than male physicians, and further excel in patient-centered communication.^16^ In a survey of medical students as well, female students had a higher empathy score than male students.^17^ From these investigations, we believe that it would be beneficial for patients if hospitals and medical institutions develop an environment and support structure so that female physicians can continue to work.  Our survey also showed that female students accepted the burden of childcare and housework as inevitable, suggesting that their career development will be affected by the environment they choose. Even in other countries, it has been pointed out that selection of a specialized field by female medical students is influenced by their surrounding circumstances, and there are no consistent career advancement programs or policies.^18^^,^^19^  These reports also suggest that it is necessary to develop a support environment that is standardized internationally and develop medical education programs to some extent, although there are differences in cultures and features of each country and region.　Moreover, urging women to face obstacles and become involved in the effort to eliminate them will be important when discussing their academic career development.^20^^,^^21^ While support from their male counterparts and institutions must increase, voluntary actions and a change in mentality of female students need to be promoted through medical education to prevent them from passively waiting for the situation to change.

A limitation of the present study was that the medical students were those who attended a single medical school in Japan. There is a need to take into account differences in regional characteristics of medical schools and various backgrounds of medical students.

## Conclusions

Although this research revealed the results in Japan, other countries also consider as important making an environment to support physicians to continue working.^4^ Childcare support for doctors regardless of gender is important. However, some male and female students who responded to our questionnaire said that they did not wish to have children in the future. There are physicians who have no children by choice or because of some health problems regardless of gender. It is a personal choice, and not something to be criticized. People with no children also need support for their career development. Physicians improve their skills through continuous training and education based on their own abilities and consistent with the needs of the society. Many institutions focus on childcare support for female physicians when addressing career development issues. However, the issues concerning childcare support should be addressed as a matter of course. It is desirabl for medical institutions to maintain a strong intention to overcome various problems faced by physicians, and to develop an educational system that emphasizes that awareness.

### Conflict of Interest

The authors declare that they have no conflict of interest.
